# A qualitative exploration of the impact of healthcare pre- and post-death on bereavement experiences

**DOI:** 10.1017/S1478951525100837

**Published:** 2025-10-23

**Authors:** Brendan Myhill, Kristin Bindley, Michelle DiGiacomo, Lillian Zhang, Michael Dash, Ivy Gough, Ghauri Aggarwal, Anthoulla Mohamudally, Lauren Stewart, Edward Lie, Jamie Parker, Nancy Huynh, Megan Ritchie, Catherine Taylor, Jessica T. Lee

**Affiliations:** 1 Concord Centre for Palliative Care, Concord Repatriation General Hospital, Sydney, NSW, Australia; 2 Sydney Local Health District, Sydney, NSW, Australia; 3 Improving Palliative, Aged and Chronic Care through Clinical Research and Translation (IMPACCT), University of Technology Sydney, Sydney, NSW, Australia; 4 University of Sydney, Sydney, NSW, Australia; 5 Department of Palliative Care, Royal Prince Alfred Hospital, Sydney, NSW, Australia; 6 Consumer Co-investigator, Sydney, NWS, Australia

**Keywords:** Bereavement, death, communication, hospitals, family, end of life

## Abstract

**Objectives:**

Many factors are known to influence experiences in bereavement. With a growing focus on public health approaches to bereavement support, it is important to further understand factors which healthcare workers (HCW) can influence regarding bereavement experiences for families. The study aim was to describe the experience of people bereaved following a death in Sydney Local Health District (SLHD), with particular focus on people’s awareness and experience of available supports and the perceived impact of healthcare interactions on bereavement experiences.

**Methods:**

The study used semi-structured qualitative interviews (n = 15) to explore the experiences of bereaved people. These were recorded, transcribed, and analyzed using a Reflexive Thematic Analysis approach.

**Results:**

Themes were generated showing the ways in which healthcare and bereavement experiences are mediated by personal interactions; that information and its delivery are central to shaping experiences; and the impacts of healthcare and government system issues on experiences of care and access to support. Attention to these factors may positively impact end-of-life care and subsequent bereavement experiences.

**Significance of results:**

It is illuminating to consider the results in light of proposed public health approaches to bereavement. Our findings assist in understanding the role that HCWs have in supporting preparation for death, providing care with the potential to prevent negative bereavement outcomes, and offering short-term bereavement support. This is key in planning models that acknowledge the essential role HCWs play within public health approaches to bereavement support. Findings can inform education and training in healthcare, with a focus on approaches that affirm dignity and positive relationships, ensure sensitive and timely information provision, and enhance skilled communication. Recommendations can support policy and system improvements to enhance bereavement outcomes.

## Introduction

Grief is a natural response to loss, and in the case of bereavement, loss following death. Grief is a multifaceted experience that incorporates emotional, physical, social, cognitive and psychological responses (Penny and Relf 2017; Stroebe et al. 2007).

Numerous factors influence bereavement experiences, with the risk of complexity potentially heightened by the relationship, gender, attachment style, mental illness, bereavement history, low acceptance of impending death, cause of death, whether or not the death was sudden or unexpected, place of death (Lobb et al. 2010; Neimeyer and Burke 2012), a pessimistic world view (Tomarken et al. 2008) and social stressors (Lobb et al. 2010; Neimeyer and Burke 2012; Stroebe et al. 2007). Protective factors include the bereaved person’s capacity to draw on past experiences of loss survival, connections with family and community, ability to identify internal and external strengths, capacity to reconstruct meaning and personal identity post-loss, and availability of practical support (Boerner et al. 2013; Neimeyer and Burke 2012). Healthcare workers (HCWs) can influence factors that might shape bereavement experiences, including quality End-of-Life (EOL) communication, emotional, psychological and practical support (Durepos et al. 2019; Mah et al. 2022; Morris et al. 2020; Wen et al. 2022) and Advance Care Planning (Durepos et al. 2019). The COVID-19 pandemic was known to have had impacts on grief experiences. A longitudinal survey of bereaved people in the United Kingdom suggested higher than expected levels of Prolonged Grief Disorder (PGD). The setting of the death, and whether the death was expected were factors which influenced experiences, while feeling well supported by HCWs after the death was associated with reduced levels of PGD symptoms (Harrop et al. 2023).

A public health orientation involves a tiered approach to bereavement support; recognizing that most people will manage bereavement with the support of family and community, and information about grief and available supports (Aoun et al. 2015, 2012; Wales 2021). A moderate-risk group may need additional non-specialist support, while a small but significant proportion at risk of complex outcomes might require specialist support, given approximately 10% of bereaved people may experience PGD. (Aoun et al. 2015; Lundorff et al. 2017). Additionally, people can encounter complexities in bereavement which may necessitate support, including financial disadvantage, strain and insecurity (Hudson et al. 2011; Roulston et al. 2017; Stroebe et al. 2007); depression, anxiety and other mental health concerns (Stroebe et al. 2007); experiences of trauma (Ganzel 2018; Stroebe et al. 2007) and increased risk of mortality (Stroebe et al. 2007).

Understanding the work of HCWs in the context of the public health approach is essential, given advocacy for transitional models of care which acknowledge the necessity of institution-based care from healthcare services and “a bereavement-conscious workforce” alongside “health and social care systems that centre bereavement as an inherent element of the duty to care.” (Lichtenthal et al. 2024, 273).

Increased knowledge about the HCW role in preparing for death, providing anticipatory bereavement care and short-term bereavement support is vital, necessarily informed by the lived experience of bereaved people.

### Aim

This study sought to describe the experience of people bereaved following a death in Sydney Local Health District (SLHD), including their awareness and experience of available supports, and exploration of the impact of healthcare interactions on bereavement.

## Methods

### Design, ethical approval and recruitment

This article reports on a qualitative study which constituted one element of a broader study, first involving a quantitative survey of bereaved people. This survey had the same aims as this qualitative sub-study, with the addition of exploring whether care was provided in line with the principles of trauma-informed care, as well as experiences of Advance Care Planning. Recruitment for interviews was undertaken at the conclusion of the survey. The study was conducted in a health district in a capital city, including three acute care hospitals, a palliative care unit and care at home through community health services.

During the study period (September 2021–November 2022) 2,600 people died within SLHD. Following exclusions, 1,929 surveys were sent to potential participants (see Table 1). Of the 293 participants (15.7% response rate) who completed a survey, 83 provided contact details offering to take part in an interview. Fifteen participants were selected to be interviewed. This sample size is guided by the concept of information power, where the more information the sample holds relevant to the study aims, the lower the number of participants needed (Malterud et al. 2016). Information power depends on the study aims, the specificity of the sample, dialogue quality and analysis strategy (Malterud et al. 2016). The first 5 participants were recruited sequentially, and then subsequent participants were included purposefully, guided by the concept of information power to ensure variety of characteristics including gender, relationship, setting and time since death.

**Table 1.** Inclusion and exclusion criteria

Inclusion criteria	Exclusion criteria
The main contact person/Next of Kin (NOK) for a patient who died in the care of SLHD (in a hospital, or at home under the care of the community nursing service)	Participants with incomplete contact details
The death occurred in the preceding 2–15 months	Participants with overseas contact details
The patient who died was 18 years or older at time of death	The patient who died had contact with only an outpatient clinic
Participants were over the age of 18	The place of death of the deceased is not clearly recorded
Participants were able to understand and speak English at a level sufficient for participation	

The interviewer was a male social worker experienced in end-of-life care (EOLC) and early bereavement. This skillset facilitated sensitive and open exchange, enhancing dialogue quality. Participants were not known to the interviewer, and a semi-structured interview guide was followed.

A multidisciplinary group of investigators developed the interview guide based on study objectives (topics shown in Table 2). The team held experience in EOLC, bereavement and research and included a consumer investigator. The study was approved by the Concord Repatriation General Hospital Human Research Ethics Committee (reference 2022/ETH01743).

**Table 2.** Interview topics

Background to the participant’s relationship to the deceased.
Exploration of the way HCWs communicated with the participant around the time of the death, interactions with HCWs, levels of consultation and information provision as the patient approached EOL.
Experience of Advance Care Planning conversations.
Practical arrangements the participant had to make following the death, and any guidance they received.
Participant expectations of support they might receive from the health service after the death, experience of receiving the support and whether expectations were met.
Exploration of their experience of grief, conversations with HCWs about grief and what to expect, any supports they found helpful or unhelpful, and religious, spiritual or cultural aspects of grieving.
Self-report of ways in which experiences of the care in the health system may have impacted on their grief.
Invitation to offer advice to HCWs on how they can make bereavement experiences better for families.
Space for any other relevant topics the participant wished to raise.

Of the 15 interviews, 12 interviews were undertaken face-to-face at a location chosen by the participant, and three were conducted by telephone. Interviews were audio recorded and transcribed. As analysis occurred concurrently with interviews, coders were able to determine that a level of saturation of themes was reached when new themes were created infrequently (Guest 2006), and interviews were ceased.

## Analysis

Reflexive Thematic Analysis (RTA) (Braun and Clarke 2020) was employed, underpinned by a constructionist lens. Constructionism is a congruent approach to explore culturally, socially and spiritually contextualized experiences of care, grief and bereavement (Schwandt 2005). Analysis began with listening to interview recordings, re-reading transcripts, and noting impressions. Inductive coding was then undertaken by two team members (BM, LZ) who both coded 3 transcripts to compare coding and then divided the remainder. Preliminary coding was presented to an investigator team sub-group, to inform agreement regarding the initial coding approach. NVivo 12 software was used to support coding (Lumivero 2017), with the codebook evolving iteratively as understanding deepened. Coders met regularly to discuss progress with codes, their meanings and to question assumptions. The positioning of each coder assisted this process, being from social work and medical backgrounds, respectively. Initial themes were generated in discussions between coders and enriched by each team member’s unique experience, perspective, and values. The broader investigator team met and reached consensus on themes and implications from the data.

To enhance rigor, interview participants who indicated interest received a summary of preliminary findings, and invitation to respond (Thorne et al. 1997). Two participants replied that they felt the summary reflected their interview appropriately and required no changes.

## Results

Participant characteristics are displayed in Table 3. Of the 15 participants interviewed, 10 were female and 5 were male. Seven participants reported that they were the spouse of the deceased, 7 an adult child, and 1 was a sibling. The majority were born in Australia, with 2 born in the United Kingdom and one in Greece. The age range for participants was 40–49 (N = 3), 50–59 (N = 5), 60–69 (N = 4), 70–79 (N = 3). Time since the death (at time of interview) was 4–6 months (N = 2), 6–12 months (N = 10) and longer than 12 months (N = 3). For all participants English was the main language spoken at home. None of the interviewees were bereaved following a sudden death. All deaths included experiences of EOLC provided by HCWs, and occurred across a range of settings including hospital wards, intensive care units, a specialist palliative care unit and at home with the support of community palliative care.

**Table 3.** Participant characteristics

Gender	Female	10
	Male	5
Relationship to the person who died	Partner/spouse	7
	Sibling	1
	Child of the deceased	7
Country of birth	Australia	12
	United Kingdom	2
	Greece	1
Main language spoken at home	English	15
Age	40–49	3
	50–59	5
	60–69	4
	70–79	3
Place of death (as noted in interview)	Home	3
	Hospital ward	7
	Intensive care unit	3
	Palliative care unit	2
Interview format	In person	12
	Phone	3

The research team generated three main themes, namely that healthcare and bereavement experiences are mediated by personal interactions and honoring relationships, information and its delivery are central to shaping experiences, and healthcare and government system issues impact experiences of care and access to support. These themes are displayed, along with sub-themes, in Table 4. A list of codes used in NVivo is available on request from the authors.

**Table 4.** Themes and sub-themes

Theme	Sub-themes
Healthcare and bereavement experiences are mediated by personal interactions and honoring relationships	The centrality of relationships to the experience of healthcare and bereavement
	The importance of presence, respect and dignity
	Honoring the relationship, including into bereavement
Information and its delivery are central to shaping experiences	Information is needed to help to make sense of the story
	Preparation is key, access to information can facilitate meaningful interactions at EOL
	Information helps with understanding grief and the transition to early bereavement
	Honest, direct communication is associated with trust
Healthcare and government system issues impact experiences of care and access to support	System issues impact communication, care and access to support
	System issues shape perceptions of care, and families carry the burden when systems don’t work well
	A more systematic approach to information provision and support in early bereavement is required

### Healthcare and bereavement experiences are mediated by personal interactions and honoring relationships

While reflecting on experiences of care pre- and post-death, participants shared insights into the personal nature of healthcare relationships and their impacts, often commenting on interactions with individual HCWs. Relationships that affirmed the dignity of the dying person and conveyed respect to the family were highlighted. A bereaved husband reflected:

“the nurse put my wife’s – did her hair and put it up in a bun and everything like that. Although my wife was effectively unconscious, the nurses used to talk to her as if she was there and conscious, and, you know, they’d walk in and, “hi, (name), I’ve just come on shift, my name’s (nurse’s name). I’ll be looking after you for the next 12 hours”…that added to the dignity of the process.” (bereaved husband, study ID 126)

Where participants reported that relationships were not respected, this shaped perceptions of care. This included relationships between HCWs and patients, and attitudes shown towards relationships between patients and their supports. One participant commented on a sense of disrespect in language used by a HCW when family sought a visiting exemption during COVID-19 restrictions:

“these were the words ‘the visit would be only beneficial to you, not the patient’ That is unacceptable.” (bereaved daughter, study ID 166)

The importance of the visible presence of familiar HCWs was noted, seen as a crucial aspect of support. The absence of known HCWs may be interpreted as lack of support. Following the expected death of her husband one participant reflected:

“I don’t know whether it was the change of shifts or what it was but there just was not one familiar face there…not one person, one nurse, that we had dealt with or had been looking after (husband’s name) that we felt – there was no one” (bereaved wife, study ID 4)

Post-death contact from HCWs in the form of personalized letters, cards, funeral attendance or follow-up calls were ways that relationships were honored, conveying a sense of ongoing care. Lack of follow-up when expected, especially after a long relationship, could be seen as disrespectful.

“I thought he could have given me a call, seeing that he’s been my GP for, wow, 50 years, maybe more…A long relationship. Yeah, I was a bit disappointed with that.” (bereaved wife, study ID 115)

### Information and its delivery are central to shaping experiences

Participants outlined the ways in which information and communication shaped their experience of EOLC, early bereavement and grief. Families who had been unable to witness changes in a patient’s condition or lacked regular access to HCWs for information and updates described unresolved issues after the death. This included a disruption in the narrative in making sense of the person’s death. One participant reflected on being unable to see her father prior to his death due to COVID-19 restrictions:

“There was this hole if you like. Dad went there, he passed away and that was the end of the story…although I was speaking with the doctor – I mean there’s only so much we can cover. After that it was all over. I suppose it didn’t really matter anymore. What did matter is the unknowing – the unknown of the week” (bereaved daughter, study ID 166)

Although this scenario referred to a time of visiting restrictions during the pandemic, it highlights the experience that people may face we they have insufficient access to information or are unable to visit. This “hole” carried through into the participant’s experience of grief and ability to process and make sense of the death and loss. What she wanted was a chance to meet the care team, ask questions, have a description of her father’s progression and deterioration; to fill the gaps between his admittance and death, and make sense of the story.

There were links to negative consequences, such as guilt or regrets, for the bereaved person when desired information was not provided in advance. This included education regarding the dying process, symptoms at EOL or prognosis. Participants reflected on lost opportunities for meaningful interactions around the death event including being present at the time of death, having spiritual needs addressed, or involvement in rituals such as washing the deceased’s body. One participant recalled a perceived missed opportunity to communicate with her husband because consequences of medication changes were not explained:

“it would have been nice to have that last conversation…I guess I wished that someone had said ‘before we do this, do you want to have a chat with him?’ because he’s probably not going to talk much after this or be awake much” (bereaved wife, study ID 10)

This emphasized the importance of preparedness, and linkage between lack of deliberate anticipatory communication and regrets for family members; contributing to their experience of grief.

Information provision in advance about grief was suggested as helpful. This included understanding the way people can grieve differently, and what to expect in grief. One participant reflected on the impact of grief education in understanding his young daughter’s response:

“The social worker was quite good, helping me understand the different reaction of myself compared to my daughter.” (bereaved husband, 126)

The power of information in normalizing grief experiences and assisting the transition into bereavement was evident. Information on the practicalities of early bereavement was also seen as beneficial for guiding funeral planning and death administration, including death notifications and dealing with finances.

The way communication occurred strongly influenced participants’ perception of care at EOL and sense of trust in HCWs. Participants discussed the importance of communication that was clear, honest and direct. This was associated with trust in the care team, even when news was difficult to hear. One participant recalled a conversation about his wife’s limited prognosis when in an Intensive Care Unit:

“It was scary, but I appreciated it. I felt I trusted what he said because he was very direct…in hindsight, I preferred that more direct approach. I think that better helped myself and the family to be prepared for what was coming.” (bereaved husband, study ID 126)

Mistrust can develop where families feel that they do not have access to all information, or perceive a lack of transparency, which was especially important in preparing for transitions between care environments. One person reported feeling she had not been fully informed about what to expect with a move to a palliative care unit and associated limits of care. This impacted relationships with HCWs:

“I felt that there was a little bit of mistrust there because I just didn’t feel like they gave us the right information or the whole story” (bereaved wife, study ID 4)

### Healthcare and government system issues impact experiences of care and access to support

Participants shared insights into the way systems issues impacted EOL and early bereavement experiences. The personal impact of system issues included delayed implementation of visiting policies during the pandemic, with subsequent disruptions to access to the dying person. One bereaved daughter reflected:

“the government announced that, yeah, visitors could come in, but we got there, and the staff knew nothing about it…They said, ‘oh, it’ll have to come down from the manager in this particular area’. They said it could be a week before we receive the news.” (bereaved daughter, study ID 17)

Families were also impacted by intra-facility communication processes and resourcing. Some reported significant challenges navigating systems to make appointments, access information or psychosocial support. Communication between HCWs influenced perceptions of care, which included distress for family members mediating decision-making and information-sharing between care teams:

“the nurses were still trying to do things, but it was conflicting with what Palliative Care was trying to do…My daughter caught the brunt of that one night where she had to be very, very forceful that Palliative Care be called.” (bereaved husband, study ID 35)

At an interservice level, systems for health providers to share information were lacking, including death notifications. This impacted family perceptions of care at EOL and the ability of HCWs to offer post-death support. One participant was surprised that her husband’s GP was not aware of his death in hospital:

“He said ‘I had no idea, no idea.’ And we just couldn’t believe that the systems don’t talk to each other … that there’s not some sort of alert sent out.” (bereaved wife, study ID 117)

A systematic approach to providing information and support in early bereavement was indicated. Participants expressed a need for practical information to guide funeral planning and death administration tasks. Some participants reported not receiving any information or support post-death. A younger widow wondered what navigating bereavement would be like for others:

“like people that don’t have support or family, if they get the – well, the no attention that I got afterwards, I guess that would be incredibly hard for them” (bereaved wife, study ID 10)

Participants indicated a lack of awareness of what bereavement information or support should be expected from healthcare services, and inconsistent experiences of support provided after a death. When bereavement support and information was received, some indicated a sense of this being unplanned, or due only to their relationship with the HCW.

“I’m not sure if I would’ve had that conversation if there hadn’t been belongings to pick up. I don’t know if she or anyone else would’ve rung me, would’ve contacted me…it felt a bit sort of coincidental” (bereaved son, study ID 140)

Participant responses indicated a need for systematized information about what support could be expected following a death. Frustrations were evident with increased burdens in dealing with government agencies and systems that don’t communicate to share death notifications.

## Discussion

Findings emphasize the significance of personal interactions in healthcare, and the ways they inform bereavement experiences. This resonates with recent survey findings that compassionate care that conveys empathy and underscores a human connection can help families in dealing with loss (Morris et al. 2020). Personalized healthcare relationships and support have also been found to be necessary for successful empowerment and advocacy in EOL (Reed et al. 2018). Additionally, bereaved families report initial post-death follow-up and support from HCWs who were involved pre-death to be helpful (Harrop et al. 2016; Morris et al. 2020), with potential for a compounded sense of loss associated with the cessation of healthcare service support in bereavement (Harrop et al. 2016; Holtslander et al. 2017; Stajduhar et al. 2010; Tabler et al. 2015). This emphasis on relationships is echoed in our findings and aligns with work on the importance of dignity in care (Chochinov 2022).

We identified a key relationship between access to information at EOL and a person’s ability to later integrate experiences in bereavement. People assimilate the story of a death in ways that makes sense of the story of their lives (Neimeyer et al. 2014), making the ability to access to information, and witness changes key. Participants expressed a need for open and honest communication in EOL. It is increasingly established that families want honesty about prognosis and what to expect at EOL. A recent review found this is the case in places and cultures where providing explicit information was not seen as usual practice (Morris et al. 2020; Welsch and Gottschling 2021). A small proportion however may manage better by not having explicit information (Welsch and Gottschling 2021), making consideration of information and communication preferences essential.

Open discussions with patients and families can allow sensitive information provision about prognosis, and EOL symptoms, it also allows practical preparations for dying or visits with a spiritual advisor which can reduce distress (Mah et al. 2022). Information provision can also promote connectedness, allowing family time together and saying goodbye (Mah et al. 2022). The association between lack of preparedness and complex bereavement experiences is recognized (Lobb et al. 2010; Neimeyer and Burke 2012). A significant association has been found between death preparedness and bereavement outcomes, particularly conjoint cognitive and emotional preparation (Wen et al. 2022). Preparedness can be supported by quality HCW communication including prognostic communication, advance care planning, and psychological support (Durepos et al. 2019; Mah et al. 2022; Wen et al. 2022). Our study also demonstrates the benefits of HCWs providing information about grief and bereavement. Educating about emotions and grief responses is a useful HCW skill that promotes mental health and well-being after death and adjustment in bereavement (Durepos et al. (2019).

Our findings identify the ways in which system issues impact families throughout illness and into bereavement. Australian research highlights the challenges bereaved people face with bureaucratic processes and rigid organizational policies and protocols (Bindley et al. 2021; Blackburn and Bulsara 2018). Bereaved carers encounter structural burden and stress related to navigating complicated systems, which is amplified for carers who are structurally vulnerable; meaning their positionality places them at increased risk of experiencing disadvantages due to dimensions such as age, socioeconomic status, and ethnicity (Bindley et al. 2021). Such individuals are at particular risk of falling between the cracks and entrenched inequity where systems do not operate cohesively (Stajduhar et al. 2019).

Our findings also show a need for HCWs to provide deliberate communication so families know what bereavement support to expect from healthcare services and related systems. Clear, consistently applied guidelines can assist HCWs and institutions to know their roles and responsibilities in bereavement care, yet there are no broader, national Australian standards for bereavement support currently. Bereavement care is seen as integral to palliative care, however even within palliative care services, bereavement support is known to be variable. One Australian survey of bereaved people found follow up support was not consistently provided in line with available guidelines (Aoun et al. 2017; Hudson et al. 2012), and another found variations in the level of bereavement support provided by palliative care services (Korbel et al. 2019). A European survey showed a significant minority of palliative care services provided no bereavement care, and 66% reported support was not based on formal guidelines (Guldin et al. 2015). A recent scoping review argued that palliative care bereavement services need to more clearly define their primary purpose and how that meets the needs of bereaved people and expectations of care (Jurgens et al. 2025). Practice beyond palliative care settings is less clear. Greater clarity at a policy level is required so that both HCWs and families know what to expect.

While some participants spoke about challenges of care during the COVID-19 pandemic, these were observed as examples of experiences that patients and family members often have in healthcare. This included challenges with communication, access to information, the ability to visit and witness changes in a patient’s condition, and impacts of system dysfunction, all of which may have had more impact on patients and family due to the pandemic. Our recommendations align with those resulting from research which followed the COVID-19 pandemic, which highlight the need for HCWs to show respect to family relationships, provide information and quality communication, promote the ability for contact and visiting prior to death, and the need for consistent follow up by care providers (Harrop et al. 2023; Selman et al. 2022; Torrens-Burton et al. 2022).

It is illuminating to consider our results in light of public health approaches to bereavement and the proposed transitional model. This model identifies that bereavement care is part of the continuum of care delivered by clinical teams, underpinned by the principle of non-abandonment, with a responsibility to address health related suffering in both preventative bereavement care and follow up (Lichtenthal et al. 2024). Our findings affirm the centrality of HCWs in enabling a transitional model, and assist in mapping approaches to achieve this; outlined below.

### Recommendations for clinicians and policy makers

Care approaches that acknowledge the human nature of healthcare interactions are recommended and can be promoted by education on dignity-conserving care. The power of timely information and honest, open communication is evident. Training in communication is central in supporting HCWs to facilitate conversations in a sensitive manner, guided by a families’ preferred level of information. Education must highlight the need for information and support during key transition points, as this can reduce or prevent distress at these times of increased uncertainty and possible distress for families.

Deliberate anticipatory communication can assist a family’s preparation for an expected death, which is known to assist in bereavement especially when covering emotional, pragmatic and informational domains. This includes, where possible, advice of when death is approaching. Policies and practices which facilitate family presence at EOL where desired are needed, along with offers of post-death consultations to fill information gaps. These approaches facilitate witnessing and information provision to support families in building a coherent post-death narrative. Consideration must also be given to tailoring information to individual needs. This is particularly important for families from Culturally and Linguistically Diverse (CALD) communities, First Nations peoples and other priority populations known to face multiple structural disadvantages, barriers and vulnerabilities in Western health systems (Khatri and Assefa 2022; Nolan-Isles et al. 2021).

Continuity in care and follow up after an ongoing relationship is important. Participants saw personalizing follow-up contact as vital, whether through follow-up phone calls, or cards and letters tailored in meaningful ways to avoid the sense of being a “form letter.” Contact offering support and information in early bereavement can also bring the healthcare relationship to a close respectfully.

Systems improvements are necessary to ensure consistent provision of bereavement information and support, including clearer guidelines for health services on bereavement responsibilities. Such guidance should facilitate further integration of health, government and social care services, to address structural barriers and aid cohesive bereavement care. This includes functional systems that reduce the burden of death administration tasks such as the Australian Death Notification Service, or the United Kingdom’s “Tell Us Once” service. Future work should expand the range of connectable agencies and services. Our results show the need for practical information on navigating early bereavement, and for systems in health services ensuring consistent information and support provision, to enable linkage with other relevant care systems as healthcare-based support decreases over time.

### Limitations and future research

Findings have been limited by a lack of inclusion of CALD and First Nations’ voices. This study could have been enhanced by actively seeking to include participants who are vulnerably positioned and collecting information relating to these characteristics. Interviews included some participants who were bereaved during the COVID-19 pandemic, which impacted their experience of care systems and supports; this was considered in our examination of findings and in our discussion. Analysis could have been strengthened by collection of dates of death which could have been correlated with COVID visiting restrictions, and cause of death. Topics for future research include exploration of the timing, content and structure of information provision at EOL and in bereavement, including preferences and needs of CALD, First Nations and socio-economically vulnerable communities.

## Conclusion

Our findings show that experiences at EOL and in early bereavement are shaped by personal interactions with HCWs; access to and delivery of information and by the functioning of healthcare and government systems. Attention to these factors can positively impact EOLC and subsequent bereavement experiences. These findings can inform HCW education and training, policy development and system improvements. The role of HCWs in preparing families for death, providing information and support, and in providing bereavement care constitutes an essential element of universal bereavement support within the public health framework, and must be supported with clear roles and expectations in policy.
